# Mechanism of Filling and Feeding of Thin-Walled Structures during Gravity Casting

**DOI:** 10.3390/ma8063701

**Published:** 2015-06-19

**Authors:** Faguo Li, Jiao Zhang, Fenggang Bian, Yanan Fu, Yanling Xue, Fucheng Yin, Yu Xie, Yi Xu, Baode Sun

**Affiliations:** 1 State Key Laboratory of Metal Matrix Composites, Shanghai Jiao Tong University, Shanghai 200240, China; E-Mail: lifaguo@sjtu.edu.cn; 2 Shanghai Key Lab of Advanced High-temperature Materials and Precision Forming, Shanghai Jiao Tong University, Shanghai 200240, China; 3 Shanghai Synchrotron Radiation Facility, Shanghai Institute of Applied Physics, Shanghai 215600, China; E-Mails: bianfenggang@sinap.ac.cn (F.B.); fuyanan@sinap.ac.cn (Y.F.); xueyanling@sinap.ac.cn (Y.X.); 4 School of Materials Science and Engineering, Xiangtan University, Xiangtan 411105, China; E-Mail: fuchengyin@xtu.edu.cn; 5 University of Michigan-Shanghai Jiao Tong University Joint Institute, Shanghai Jiao Tong University, Shanghai 200240, China; E-Mail: yu.xie@sjtu.edu.cn; 6 School of Electronic Information and Electrical Engineering, Shanghai Jiao Tong University, Shanghai 200240, China; E-Mail: xuyi@sjtu.edu.cn

**Keywords:** aluminum alloys, thin-walled effect, solidification microstructure, casting

## Abstract

The filling and feeding of thin-walled structures in metal castings pose significant difficulties in manufacturing aerospace structural materials. Samples containing 2 mm and 5 mm thin-walled structures were designed to study the kinetics of filling. The microstructural evolution of the solidification of thin-walled structures was studied with synchrotron X-radiation imaging. The formation of dendritic networks and the isotherm profiles of samples of different thickness were examined. The experimental results showed solidification microstructures of 2 mm and 5 mm thin-walled parts containing elongated equiaxed grains and normal equiaxed grains, respectively. The filling and feeding abilities of thin-walled parts were found to depend more on the wall thickness than on the pouring temperature.

## 1. Introduction

Large-size thin-walled castings of aluminum, titanium, and nickel alloys with complex structures are widely used in the aerospace industry. The lightweighting of aeronautic designs requires the use of structural castings with even thinner walls, which are known to resist filling and feeding during the casting process. While die-casting and low-pressure casting can be used to fabricate thin-walled aluminum- or magnesium-based light alloy castings [1], most large and complex thin-walled castings of high-temperature alloys still depend on traditional gravity casting.

Increasing the pouring temperature and mold shell temperature are currently considered the most effective methods to improve the filling ability of thin-walled castings. However, recent studies have shown that these increase the probability of shrinkage defects, hot-tearing, and harm to the mechanical properties of the cast material. Studying the dynamic behaviors of the solidification process facilitates the elimination of these problems. In previous works, the focus has largely been on the factors involved in the microstructural formation of thin-walled castings; these factors include wall thickness (*W*), pouring temperature (*T_pr_*), and cooling rate (*R_c_*). *W* heavily affects the cooling rate [2,3]. Decreasing *W* usually decreases the dendrite arm spacing (DAS) and grain size (*D*_gr_) in the cast material. The amount of eutectics in the alloy has a complex dependence on the cooling rate [4]. In terms of *T_pr_*, the grain size experiences three size regimes as *T_pr_* increases: (i) relatively constant; (ii) non-linear increase; and (iii) linear increase [5].

To obtain perfect thin-walled castings, the features must be completely filled and sufficiently fed during solidification. During the casting process, thin-walled structures are subject to high rates in both cooling and dendritic growth. The flow of the alloy melt in these features is prohibited shortly after pouring because of the fast formation of the dendritic network. The micropores will form if the solidification shrinkage of the interdendritic melt is not supplemented. The referenced supplementing process is relevant to features including the morphology of the dendritic network, the density of dendritic network, and solute distribution. However, the existing studies on the filling of thin walls were based on post-casting analysis [6,7] and simulation [3,8], which cannot accurately capture the dynamic process. In the last decade, synchrotron X-ray radiography has been successfully applied to the study of metallic-alloy solidification. By this method, researchers around the world obtained invaluable information on dendritic growth [9,10,11], solute distribution [12,13], and dendritic fragment drift [14,15], as well as other phenomena. Synchrotron X-ray radiography was adopted to study the evolution of microstructure in thin-walled structures here. The microstructural evolution during solidification of the specially designed thin-walled Al–20 wt% Cu alloy samples was studied. The influences of *W* and *T_pr_* on solidification microstructures were comprehensively considered and discussed.

## 2. Experimental Section

**Figure 1:**
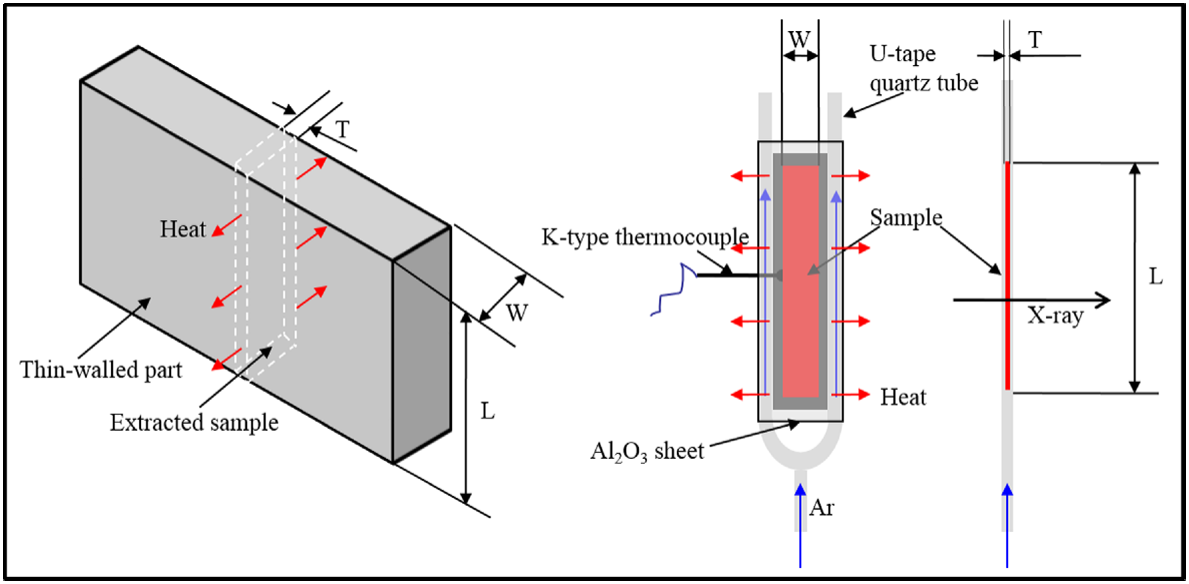
Schematic of the thin-walled sample and the cooling method. W = width, L = length, T = thickness.

According to the features of typical thin-walled castings, two sample geometries with wall thicknesses of 0.5 mm and widths of 2 mm and 5 mm were prepared. As shown in Figure 1, the 0.5-mm- thick Al–20 wt% Cu (melting point *T_m_* = 600 °C) slice is sandwiched by two Al_2_O_3_ sheets and sealed with a mixture of silica sol and Al_2_O_3_ powders (the mixture was used as a kind of high temperature glue to seal the samples), then mounted on a U-tape quartz tube. The two quartz tube branches are set along the two sides of the sample to permit cooling by passing Ar through the tube.

The X-ray radiography experiments were carried out in a self-made vacuum resistance heating furnace. Before freezing, the samples were heated to beginning temperatures (*T_B_*) of 680 °C, 700 °C, 750 °C, 800 °C, or 850 °C, to simulate different pouring temperatures and mold temperatures. After holding for 15 min at the *T_B_* to homogenize the melt, high-purity Ar gas was introduced to the quartz tube from the bottom inlet at a constant flow rate of 8.51 m^3^ h^−1^ at 0.1 MPa pressure. The cooling rate was measured by an embedded K-type thermocouple, as shown in Figure 1.

The synchrotron X-ray phase contrast imaging [16] experiments were performed at BL13W of SSRF (Shanghai Synchrotron Radiation Facility, Shanghai, China). The incident monochromatic X-ray energy used in all experiments was 25 keV. The spatial resolution of all images was 7.4 ×7.4 μm^2^ pixel^−1^. The exposure time for each frame was 0.5 s.

## 3. Results and Discussion

### 3.1. Solidification Microstructure

#### 3.1.1. Dendrite Structure

**Figure 2:**
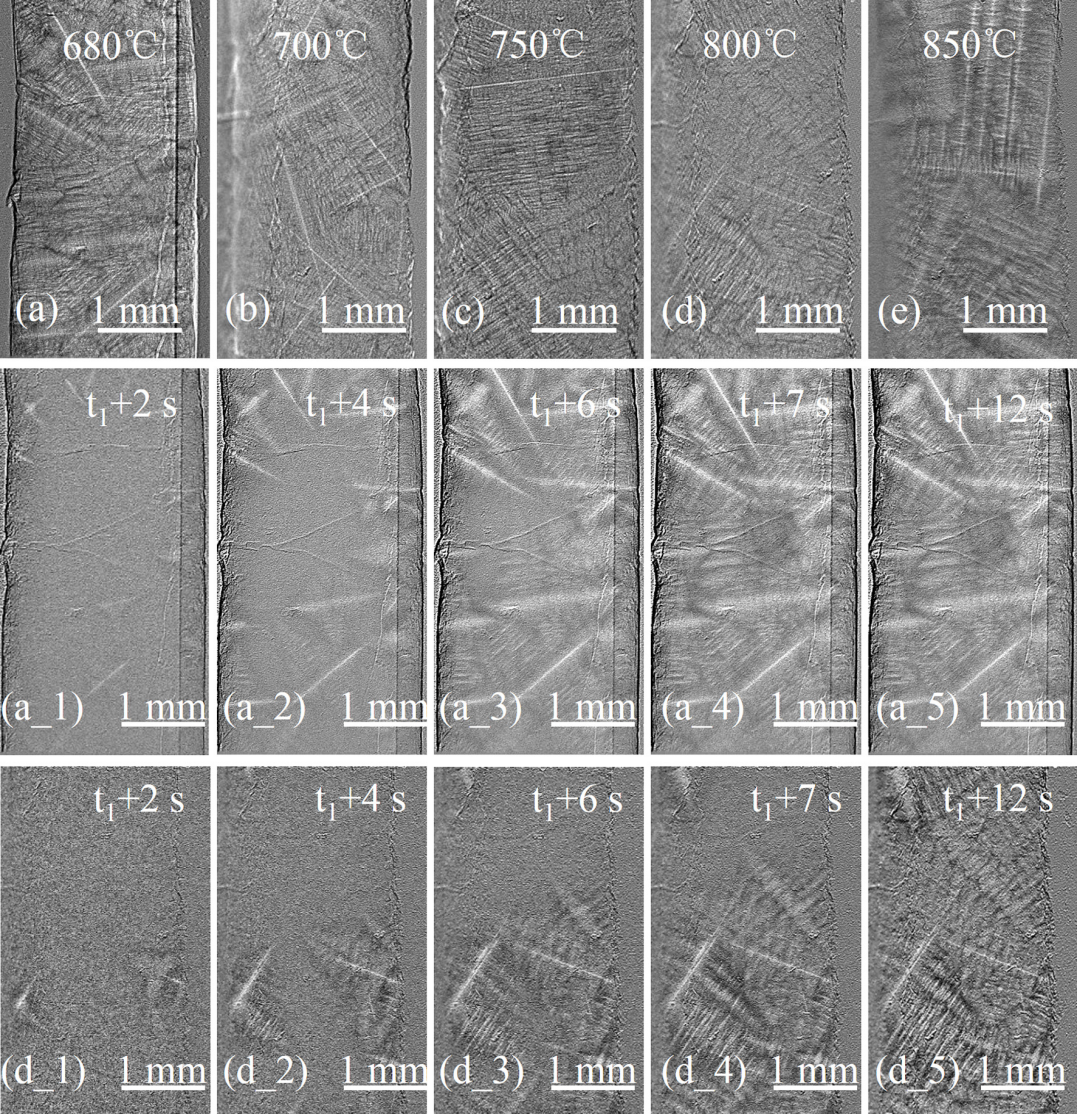
The microstructure of the 2 mm sample under different beginning temperatures. (**a**) 680 °C; (**b**) 700 °C; (**c**) 750 °C; (**d**) 800 °C; (**e**) 850 °C. Sequence of images showing the microstructural evolution of the 2 mm sample at beginning temperatures of (**a_1**–**a_5**) 680 °C or (**d_1**–**d_5**) 800 °C.

The microstructure of the 2 mm sample was composed of elongated equiaxed grains. As Figure 2a–e shows, the dendritic arms intersect to form a dense network; the eutectic phase is separated into a capillary network within the interdendritic spaces. Increasing the *T_B_* apparently does not affect this characteristic.

A sequence of images show the microstructure evolution with a *T_B_* of 680 °C (Figure 2a_1–a_5) and 800 °C (Figure 2d_1–d_5). For the 2 mm sample, the dendrites began to nucleate at the chilled edges. The number of newly born dendrites of the sample with *T_B_* of 680 °C was four times that of the sample with *T_B_* of 800 °C within *t*_1_ + 2 s. No new dendrites were found in the central area of the sample in the subsequent two seconds for both samples. After 6 s, *t*_1_ + 6 s, the volume fraction of solid (*f*_s_) in the field of view of sample with 680 °C beginning temperature was 1.8 times that of sample of 800 °C. The dendrites impinged together and formed compact network at *t*_1_ + 7 s for a low beginning temperature sample. The composition of the residual interdendritic liquid approached the eutectic point. This dense dendritic network prevented the melt from flowing, then impeded melt feeding from the neighboring areas. At this time, *f*_s_ of the high beginning temperature sample only reached 60%. When time was *t*_1_ + 12 s, the sample with *T_B_* of 680 °C completely solidified. During the solidification process, no obvious dendritic coarsening was observed.

**Figure 3:**
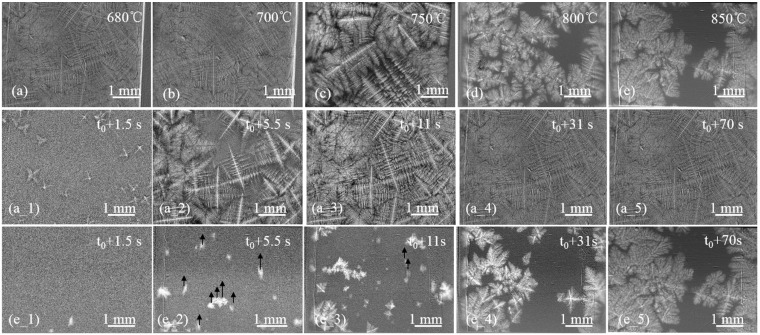
The microstructure of the 5 mm sample under different beginning temperatures. (**a**) 680 °C; (**b**) 700 °C; (**c**) 750 °C; (**d**) 800 °C; (**e**) 850 °C. Sequence of images showing the microstructural evolution of the 5 mm sample at a beginning temperature of (**a_1**–**a_5**) 680 °C or (**e_1**–**e_5**) 850 °C. Black arrows indicate the floating direction.

By contrast, the dendritic structures of the 5 mm samples under different conditions are entirely composed of equiaxed dendrites, as shown in Figure 3a–e. As the *T_B_* increases from 680 to 850 °C, the solidification microstructure diverges from the dendritic network and the eutectic phase aggregates to form an obvious eutectic zone. Under conditions of higher *T_B_*, the dendritic arms appear to be coarsening. This implies that the solidification microstructure of the 5 mm sample is strongly affected by the *T_B_*, or *T_pr_*.

For the 5 mm sample, many fine equiaxed grains appear within a short time. The number of dendrites of the sample with *T_B_* of 680 °C was 12 times that of the sample with *T_B_* of 850 °C in *t*_0_ + 1.5 s. The growth rate of dendrites of the sample with *T_B_* of 680 °C was faster at *t*_0_ + 5.5 s, too. As shown in Figure 3e_2, the equiaxed grains formed at the central area and floated up quickly in the first few seconds. Both the new dendrites nucleation and the dendrite network formation of the sample with *T_B_* of 800 °C lagged behind the sample with *T_B_* of 680 °C. When time was *t*_0_ + 70 s, the dendrite network of the sample with a *T_B_* of 850 °C finally formed.

When the sample thickness increased from 2 to 5 mm, the cooling rate decreased, the solidification rate became lower, the interdendritic space became bigger, and the number of the eutectic phase increased. According to the above analysis, the microstructure of the 5 mm sample was sensitive to the *T_B_*, unlike the microstructure of the 2 mm sample.

#### 3.1.2. Grain Size

The grain size (*D_gr_*) permits the quantitative evaluation of the solidification microstructure and material properties of a cast alloy. Table 1 shows the dependence of the cooling rate and grain size on the *T_B_*. The grain size of the 2 mm sample increases with increasing *T_B_*, in agreement with the conclusions of ref. [4,17]. However, the grain size of the 5 mm sample first increases and then decreases with increasing *T_B_*. As our previous work stated, the newly born dendrites have a lower density compared to the original liquid alloy [15]. The equiaxed dendrites floated up, nucleating in the melt. The Cu atoms were rejected from the solid phase during freezing, and the Al-enriched solid dendrites drifted away, but the Cu component was left and concentrated in the viewing area. In a liquid Al–Cu alloy, the zone with high Cu concentration has strong X-ray absorption capability, which leads to comparatively lower brightness than other areas. According to the brightness, the solute distribution can be distinguished approximately, as shown in Figure 3. This created a situation in which the liquid temperature of the melt was always less than the actual temperature, thus restraining the growth of dendrites. Finally, a bulk eutectic area formed.

This suggests that a higher pouring temperature, equivalent to the increased *T_B_* conditions here, induces the coarsening of the microstructure of thin-walled castings or the enrichment of the eutectic phase, both of which seriously reduce the performance of castings [18,19].

**Table 1:** *R_c_* and *D_gr_* of different beginning temperatures (*T_B_*) for the two geometries of thin-walled samples.

*T_B_*, °C	2 mm		5 mm	
	*R_c_*, K s^−1^	*D_gr_*, mm	*R_c_*, K s^−1^	*D_gr_*, mm
680	2.48	1.04 ± 0.30	1.68	1.18 ± 0.28
700	2.08	1.29 ± 0.16	1.04	1.33 ± 0.30
750	1.49	1.41 ± 0.29	0.69	1.45 ± 0.40
800	1.16	1.58 ± 0.20	0.50	0.80 ± 0.10
850	0.87	1.64 ± 0.20	0.33	0.75 ± 0.10

### 3.2. Thermal and Solute Fields

Relevant data were extracted from the photographic results to study the morphology selection mechanism of the thin-walled structures. The microstructure morphology was found to mainly depend on the distribution of thermal and solute fields. As mentioned above, the solidification microstructure of the 2 mm samples contained elongated grains. However, the solidification microstructure of the 5 mm samples contained normally shaped equiaxed grains. Samples were extracted from the thin-walled structures, in which the heat dissipation existing in the experiments along the direction perpendicular to the samples was presumed to be equal to zero. With the vacuum environment of the experimental setup, convective heat transfer on the samples’ surfaces can be set to zero. The Ar gas cooling on both sides of the samples similarly renders the radiative heat transfer to the sample surface negligible.

Notably, the series of photographic data shows the changing tendency of the thermal effects on the solidification times of structures of different thicknesses; in other words, higher *T_B_* correlates to larger effects on thicker structures. Therefore, the thermal gradient within the sample becomes the dominant factor in the evolution of the dendritic network.

**Figure 4:**
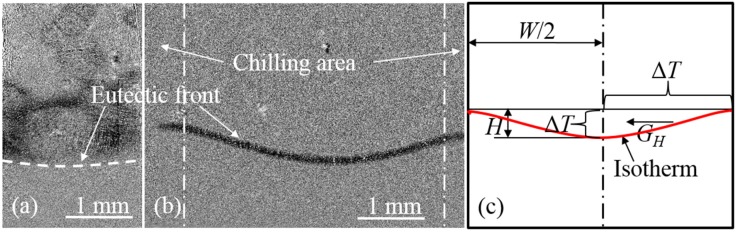
The eutectic front of the two geometries of thin-walled samples: (**a**) 2 mm sample; (**b**) 5 mm sample; (**c**) Schematic of symmetrized isotherm.

Usually, the profile of the eutectic front is considered to be a living isotherm. In our experiments, the shape of the eutectic front is elucidated by the image subtraction method, as shown in Figure 4a,b. The concave eutectic profile indicates that the temperature at the center exceeds that of the sides, and a horizontal thermal gradient (*G_H_* > 0) exists from the side to the center. As the slope along the isotherm varies continuously, the horizontal temperature gradient at each point is different.

A simplification was made in the following discussion: all factors involved take their average values. Quantitatively, if the solidification velocity of the solid phase is *V_s_*, the concave depth of isotherm is *H*, the cooling rate is *R_c_*, and the wall thickness is *W*, then the temperature difference from the surface to center (Δ*T*) is:

(1) $$\text{Δ}T=\frac{R_{c}H}{V_{s}}$$

The average horizontal temperature gradient *G_H_* is:

(2) $$G_{H}=\frac{2\text{Δ}T}{W}$$

The time difference of nucleation between the center melt and side-wall melt *t_d_* is:

(3) $$t_{d}=\frac{\text{Δ}T}{R_{c}}$$

Table 2 shows that *G_H_* increases with increasing *T_B_*. Moreover, thinner walls correlate to smaller *G_H_* values.

**Table 2:** *R_c_*, Δ*T*, and *G_H_* of different beginning temperatures (*T_B_*) for the two geometries of thin-walled samples.

*T_B_*, °C	2 mm			5 mm		
	*R_c_*, K s^−1^	Δ*T*, K	*G_H_*, K mm^−1^	*R_c_*, K s^−1^	Δ*T*, K	*G_H_*, K mm^−1^
680	2.48	0.0405	0.0405	1.68	1.9691	0.7876
700	2.08	0.0416	0.0416	1.04	2.1956	0.8782
750	1.49	0.0433	0.0433	0.69	3.1516	1.2606
800	1.16	0.0458	0.0458	0.50	3.8519	1.5408
850	0.87	0.0472	0.0472	0.33	5.1458	2.0583

For the 2 mm sample, the *R_c_* is large, the *G_H_* is very small, and the *t_d_* is approximately 0.016–0.054 s, implying that the chilling effect affects the entire section across the sample. In addition, the growth rate of the primary dendritic arms with priority exceeds 1 mm s^−1^, indicating the quick formation of the dendritic network and the interdendritic capillary network.

For the 5 mm sample, *G_H_* increases as *R_c_* decreases. For *T_B_* of 800 °C or less, the *t_d_* is approximately 1.172–4.568 s. The melt near the sides solidifies first, and the dendrites grow stepwise toward the central region (the growth rate of the dendritic arms with priority is approximately 200 μm s^−1^). Newly formed equiaxed dendrites appear in the supercooled melt in front of the dendrite tips, and the final solidified microstructure is composed of coarse equiaxed grains. As the *T_B_* reaches 850 °C, *R_c_* decreases dramatically, *G_H_* increases dramatically, and *t_d_* reaches 15.593 s. The newly formed equiaxed grains float up as the structure solidifies. The equiaxed dendrites are α-Al phase; Cu becomes concentrated after the formation and floating of the equiaxed dendrites and during the dendritic coarsening process, which prevents the production of new grains and leads to the formation of a bulk eutectic area at the center.

### 3.3. Influence of Wall Thickness on Filling and Feeding Abilities

The limiting factor in the production of thin-walled castings is the complete filling and feeding of the thin-walled features. Increasing the pouring temperature and mold temperature is a technique widely used in the industry. With wall thicknesses of 2 mm, the dense dendritic network forming during the initial solidification seriously impedes the melt flow. Simply increasing the pouring temperature and mold temperature coarsens the microstructure and causes the aggregation of the eutectic phase, both of which harm the performance of cast alloys. Here, the solidification process of the thin-walled structures was investigated deeply to identify the factors which most greatly affect the filling and feeding process.

#### 3.3.1. Filling Pressure and Feeding Pressure

The molten alloy is well known to be subjected to the viscous force *F* and Laplace pressure *P* imposed by the mold’s inner surface during the pouring and filling processes.

In the viscous force *F*:

(4) $$F=\eta S\frac{dv}{dx}$$

where *η* is the kinematic viscosity coefficient of the melt, *S* is the contact area between two flow layers, and *dv/dx* is the velocity gradient. *η* is closely associated with the temperature; it decreases with increasing melt temperature. *F* is independent of the wall thickness.

In the Laplace pressure *P*:

(5) $$P=\frac{4\sigma }{W}$$

where *σ* is the surface tension coefficient of the melt, which is determined by the wetting relationship of the molten alloy and the mold wall. As the smoothness of the mold inner surface increases, the wettability decreases, along with *σ*. *W* is the wall thickness of the casting, as established in the Introduction. *P* increases with decreasing *W*. For thin-walled castings, properly increasing the pouring temperature, improving the quality of the mold cavity’s surface, and imposing pressure can all facilitate filling.

With the formation of dendrites, the space left for the melt decreases and the resistance force increases accordingly. As the dendrites impinge upon each other, the interdendritic space forms a capillary network. Under this condition, the flow of the melt depends on the pressure gradient in the melt; the relationship between these properties can be presented by the following equations.

When the molten alloy flows in the interdendritic space driven by a pressure *P*, the maximum flow velocity *V*_1_ can be expressed by Darcy’s law:

(6) $$V_{1}=\frac{k}{\eta f_{l}}\nabla P,$$

where *k* is the permeability of the melt; *f_l_* is the liquid fraction; and ∇*P* is the pressure gradient vector.

The velocity component of the molten alloy’s solidification shrinkage is assumed to be *V*_2_, defined as:

(7) $$V_{2}=\frac{R_{c}}{G_{H}}\beta ,$$

where *β* is the solidification shrinkage rate. This represents the minimum velocity of the melt necessary to accommodate solidification shrinkage.

According to (6) and (7), if *V*_1_ ≥ *V*_2_, microshrinkage will not form. The microshrinkage-free conditions can be obtained by combining Equations (6) and (7):

(8) $$\nabla P\geq \frac{\eta f_{l}R_{c}}{kG_{H}}\beta$$

The relative ratios of ∇*P* were calculated according to Table 2 for the two sample geometries. For the 2 mm sample, the ratios of ∇*P* were:

∇*P_680 °C_* : ∇*P_700 °C_* : ∇*P_750 °C_* : ∇*P_800 °C_* : ∇*P_850 °C_* = 1 : 0.817 : 0.562 : 0.414 : 0.301, where the subscript corresponds to the *T_B_*.

By comparison, the ratios of ∇*P* for the 5 mm sample were:

∇*P_680 °C_* : ∇*P_700 °C_* : ∇*P_750 °C_* : ∇*P_800 °C_* : ∇*P_850 °C_* = 1: 0.556 : 0.257 : 0.152 : 0.075.

The ratios between the feeding pressure of the 2 mm sample and the feeding pressure of the 5 mm sample were ∇*P*_2 mm_ : ∇*P*_5 mm_|*_680 °C_* = 28.707, ∇*P*_2 mm_ : ∇*P*_5 mm_|*_700 °C_* = 42.221, ∇*P*_2 mm_ : ∇*P*_5 mm_|*_750 °C_* = 62.868, ∇*P*_2 mm_ : ∇*P*_5 mm_|*_800 °C_* = 78.049, and ∇*P*_2 mm_ : ∇*P*_5 mm_|*_850 °C_* = 114.967, respectively.

Increasing the *T_B_* can reduce the feeding pressure to some extent, making the feeding process easier for thicker-walled structures. Decreasing the wall thickness is vital for the success of the filling and feeding processes; this effect may increase by orders of magnitude with minimal decreases in wall thickness.

#### 3.3.2. Filling Time and Feeding Time

**Figure 5:**
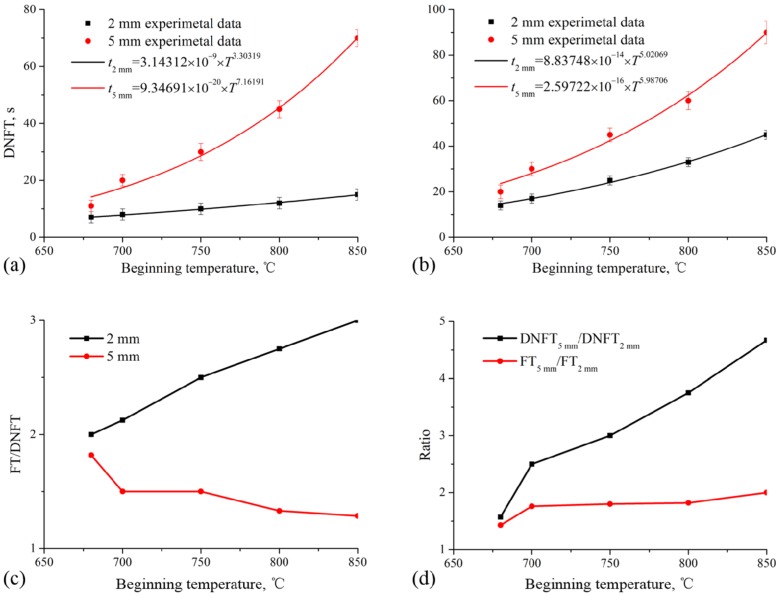
The different solidification stages’ dependence on the beginning temperature for the two geometries of thin-walled samples. (**a**) Dendrite network forming time (DNFT); (**b**) feeding time (FT); (**c**) ratios of FT/DNFT; and (**d**) ratios of DNFT_5 mm_/DNFT_2 mm_ and FT_5 mm_/FT_2_.

Filling and feeding are the two key stages for a successful casting. The filling ability of a certain alloy at the thinnest part of the mold relates simultaneously to the pouring temperature, mold temperature, and mold inner-surface properties. As all the mold-related factors are defined in a given casting, the factor left to manipulate is the pouring temperature. For high-quality castings, feeding must be deliberately considered in the design of the mold’s gating system. With metallic alloys, the solidification process can be divided into four stages: melt; coexistence of solid and liquid; eutectic phase solidification; and total solidification. From the X-ray photography results, these four stages are illustrated clearly and distinguished quantitatively. The second stage can be further divided into two parts, using the formation of the dendritic network as a separating point. This corresponds to the point at which all dendrites begin to impinge upon each other. The “melt” stage and the part of “solid and liquid coexistence” stage prior to dendritic network formation can be considered the effective filling period of the casting process. The dendrite network formation time (DNFT) is equivalent to the effective filling time. After dendritic network formation, the rest of the second stage and the “eutectic phase freezing” stage can be considered as the effective feeding period; this period ends as the eutectic melt freezes. Therefore, the feeding time (FT) is defined as the time interval after DNFT and prior to freezing. Every solidification stage is divided by the state of microstructure evolution. The main error of measurement came from the discontinuous imaging method used to present the continuous solidification process. Figure 5a shows the DNFT of the two geometries of thin-walled samples at different *T_B_* values. The experimental data can be described by the fitted exponential function *y* = *ax^n^*. The magnitude of the exponent *n* reflects the sensitivity of the DNFT to *T_B_*. When *T_B_* increases from 660 to 850 °C, the DNFT of 2 mm samples increases by only a factor of 2.14; however, the DNFT of 5 mm samples increases by a factor of 6.36, as shown in Figure 5a. Compared to the 5 mm sample geometry, the DNFT of the 2 mm sample is less sensitive to *T_B_*. Under the same conditions, the FT of the 2 mm sample increases by a factor of 3.21 as *T_B_* increases, and by a factor of 4.5 for the 5 mm sample, as shown in Figure 5b. For the 2 mm sample, generally the sensitivity of FT to *T_B_* was stronger than that of DNFT. For the 5 mm sample, the sensitivity of DNFT to *T_B_* was stronger than that of FT. This characteristic can be presented using the ratio of FT/DNFT for each sample geometry at each *T_B_*, as shown in Figure 5c. The ratio of FT/DNFT of the 2 mm sample increases with increasing *T_B_*, but the ratio of the 5 mm sample decreases with increasing *T_B_*. This indicates that even infinite increase in the pouring temperature for a 2 mm thin-walled part will not improve the filling ability.

Figure 5d shows that the ratio of DNFT between the two geometries of samples increases rapidly with increasing *T_B_*; however, the ratio of FT between the two sample geometries remains constant with increasing *T_B_*. This indicates that the filling time is sensitive to the wall thickness of castings; decreasing the wall thickness will sharply reduce the filling ability.

Therefore, increasing the *T_B_* alone cannot sufficiently improve the filling ability of thin-walled castings. Restricted by the heat resistance of the mold and the refinement of the microstructure, the pre-heating temperature of the mold cannot be raised infinitely. Castings with thin-walled parts would be of better quality if filled during the “melt” stage. In other words, thin features require rapid filling. Increasing the pouring temperature to promote the completion of the filling process before the dendritic network formation point is useful to gravity casting. However, achieving this condition is quite difficult for a large and complex thin-walled casting. Therefore, maintaining high hydraulic pressure to facilitate the flow of the melt through the interdendritic capillary network is necessary.

## 4. Conclusions

The microstructural evolution of specially designed 2 mm and 5 mm thin-walled samples was studied by synchrotron X-radiation imaging. The dendritic network formation time, the feeding time, and the isotherm profile were measured directly. Then, the effects of the pouring temperature and the wall thickness on the filling and feeding ability of thin features were discussed. The conclusions are summarized as follows:

1. The sensitivity of microstructural evolution to wall thickness declines as the thickness decreases. Thinner walls induce greater densification of the dendritic network, which shortens both the filling and feeding times.
2. Wall thickness is the vital factor determining the filling time for thin-walled parts. Infinitely increasing the pouring temperature of the molten alloy and the mold can only improve the filling of thin-walled parts to a limited extent. The results showed that the benefits to filling and feeding from increasing the pouring and mold temperatures diminish sharply as the thickness decreases. Suitable filling times under different conditions can be predicted to ensure complete filling of the mold.
3. The wall thickness also determines the feeding of thin-walled parts. A proper increase in the pouring temperature and mold temperature while maintaining a certain filling pressure can efficiently improve the feeding ability of thin-walled parts.
